# Cure of Aneurism by Compression

**Published:** 1859-07

**Authors:** 


### CURE OF ANEURISM BY COMPRESSION.

Dr. Brainard has recently succeeded in curing a popliteal aneurism by compression of the femoral artery. The compression was kept up 60 hours, when the pulsation was found to have ceased.

This is the fourth case in which Dr. Brainard has used compression for the cure of popliteal aneurism, and the first in which it has been successful. The failure in the former cases was owing to the unwillingness of the patients to bear the pain of pressure. P.

